# Molecular characteristics of rotavirus genotypes circulating in the south of Benin, 2016–2018

**DOI:** 10.1186/s13104-020-05332-7

**Published:** 2020-10-19

**Authors:** Jijoho Michel Agbla, Mathew D. Esona, Alidehou Jerrold Agbankpe, Annick Capo-Chichi, Rashi Gautam, Tamegnon Victorien Dougnon, Osseni Razack, Michael D. Bowen, Honore Sourou Bankole

**Affiliations:** 1 Ministry of Public Health, National Health Laboratory, 01 P.O. Box 418, Cotonou, Benin; 2 Viral Gastroenteritis Branch, Division of Viral Diseases, NCIRD, Centers for Disease Control CDC, 1600 Clifton Road, NE, Atlanta, GA 30329 USA; 3 Research Unit in Applied Microbiology and Pharmacology of Natural Substances, Research Laboratory in Applied Biology, Polytechnic School of Abomey-Calavi, University of Abomey-Calavi, 01 P.O. Box 2009, Cotonou, Benin; 4 Epidemiological Surveillance Service, Ministry of Public Health, 01 P.O. Box 418, Cotonou, Benin; 5 Central Clinic of Abomey Calavi, 01 P.O. Box 418, Cotonou, Benin

**Keywords:** Pediatric, Rotavirus, Surveillance, Genotypes, Benin

## Abstract

**Objective:**

Rotavirus remains the main causative agent of gastroenteritis in young children in countries that have not yet introduced the vaccine. In Benin, rotavirus vaccine was introduced late December 2019 into the EPI. This study aims to provide pre-vaccination era rotavirus genotyping data in Benin. These data can supplement data from the surveillance system of Ministry of Health of Benin which is supported by the World Health Organization (WHO).

**Results:**

Of the 420 diarrheal stool samples, actively collected in southern Benin from July 2016 through November 2018 from children under 5 years old and suffering from gastroenteritis, 167 (39.8%) samples were rotavirus EIA positive. 186 (44.3%) samples contained amplifiable rotavirus RNA detected by qRT-PCR method and were genotyped using one-step RT-PCR multiplex genotyping method. G1P[8] represents the predominant genotype (32%) followed by the G2P[4] (26%), G3P[6] (16%), G12P[8] (13%) and mixed G and P types (1%). Four samples (2%) could not be assigned both G and P type specificity.

## Introduction

Diarrhea ranks as the fifth leading cause of mortality among children under 5 years old [1]. Infectious diarrhea can be caused by microbial agents of bacterial, parasitic, viral or mycotic origin [2]. Viruses, especially rotavirus, are predominant as causative agents with more than 80% of cases [3, 4]. Globally, deaths associated with rotavirus in 2013 were estimated at 122,000–215,000 in children under 5 years of age and the largest number of rotavirus deaths occurred in sub-Saharan Africa [5].

Rotaviruses belong to the *Reoviridae* family, and possess a triple-layered icosahedral capsid enclosing a genome of 11 segments of double-stranded RNA encoding six structural and five or six non-structural proteins [6, 7]. The outer capsid proteins VP7 and VP4 define the G and P genotypes, respectively [8, 9] and rotaviruses are currently classified into 36 G and 51 P genotypes [RCWG, https://rega.kuleuven.be/cev/viralmetagenomics/virus-classification]. Although a large number of G/P genotype combinations have been reported [10, 11], the rotavirus genotypes G1P[8], G2P[4], G3P[8], G4P[8] and G9P[8] together comprise up to three quarters of human rotavirus infections worldwide [12, 13].

Rotarix and RotaTeq, have been approved and licensed in many countries. Recently, two vaccines, ROTAVAC^®^ and ROTASIL^®^ have been prequalified by World Health Organization (WHO) [14]. By the end of 2017, 32 (68%) of 47 countries in the WHO's African Region had introduced rotavirus vaccine into their national immunization programs (NIP) [15].

In Benin, rotavirus vaccine was introduced into the NIP late December 2019. It is therefore necessary to continue rotavirus strain surveillance in order to monitor rotavirus genotype post-vaccination era. This study aims to provide data for the pre-vaccine introduction period on rotavirus genotypes circulating in Benin. These data can supplement data from the surveillance system of the Ministry of Health of Benin which is supported by the WHO.

## Main text

### Methods

#### Study design, specimen handling and transport

This study is a descriptive investigation of the molecular epidemiology of rotavirus from July 2016 through November 2018 from children under 5 years with diarrhea. Diarrhea stool samples were collected from the Mênontin, Anastasie, and St. Vincent Health Centres in Cotonou and from the Central Polyclinic of Abomey-Calavi (Additional file 1: Figure S1a). Children with bloody diarrhea and those older than 5 years were excluded from this study.

All stool samples were stored at 4 °C in the laboratory or − 20 °C to facilitate further testing. All stool samples were shipped on dry ice to the CDC, USA for genotyping analysis.

#### Stool processing and nucleic acid extraction

A 10% stool suspension was prepared for each sample using phosphate-buffered saline and RNA was extracted from the suspension using either the MagMAX™-96 Viral RNA Isolation Kit (ThermoFisher Scientific, Vilnius, Lithuania) on the KingFisher™ Flex Purification System (ThermoFisher Scientific, Vantaa, Finland) or the MagNA Pure Compact RNA extraction kit on the MagNA Pure Compact instrument (Roche Applied Science, Indianapolis, IN, USA) following the manufacturer’s instructions. Prior to each of the above extraction procedures, 2 µL of 10^9^ unit/µL of MS2 bacteriophage RNA (ZeptoMetrix, Buffalo, NY, USA), were spiked into a 48 µL or 98 µL volume of 10% stool suspension to serve as an internal process control.

#### VP7 and VP4 genotyping

Genotyping was performed using reverse transcription-polymerase chain reaction (RT-PCR) to determine the G and P-genotypes. VP7 and VP4 genotyping RT-PCR was performed using a conventional multiplexed one-step amplification process with slight modifications [16]. In brief, the genotype G2 (G2-R4), G4 (G4-R2) and G9 (G9-R2) specific primers were replaced with an updated versions G2 (G2-R1: TAT GTA GTC CAT YGT ATT AGT), G4 (G4-R1: GAG CAT TCG MTA ATA MTG ATA ATA C), and G9 (G9-R3: CAG AGT ATY YTT CCA TTC HGT ATC TCC) primers. The VP7 and VP4 conventional multiplexed one-step RT-PCR genotyping product was electrophoresed on 3% agarose gels containing GelRed (Biotium, Heyward, CA, USA) for 2–3 h at 100 V and products were detected under UV transillumination or were analyzed on the LabChip^®^GX instrument (Caliper, Life Sciences, MA, USA) using the HT DNA 1 K or 5 K reagent kit (Dual protocol DNA Analysis and Quantitation) with the HT Extended Range LabChip (Caliper Life Sciences, MA, USA) as described previously [16].

### Results

#### Demographic characteristics and genotyping results

##### Distribution of the study population by age and sex

In this study, children aged from 7 to 12 months were the most represented with a percentage of 41.9% followed by those under 6 months of age (Additional file 1: Table S1). The age average was 11.3 months and the sex ratio M/F was 1.12.

All the samples were tested by EIA and the EIA positivity rate (prevalence) was 39.8% (167/420).

#### Genotyping results

##### Distribution of genotypes in Benin

**Fig. 1:**
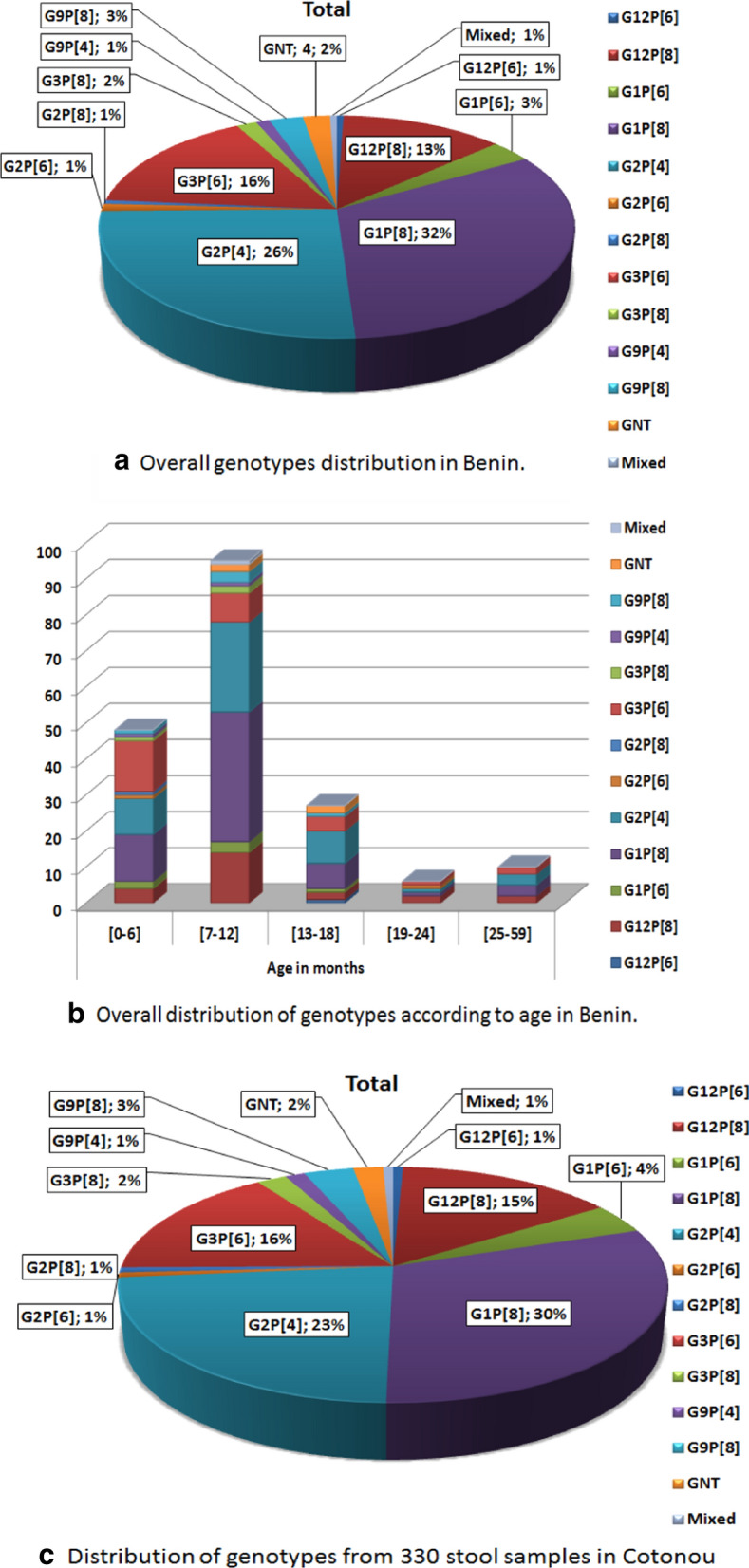
**a** Overall genotypes distribution in Benin. **b** Overall distribution of genotypes according to age in Benin. **c** Distribution of genotypes from 330 stool samples in Cotonou

Out of the 420 samples, 186 (44.3%) samples contained rotavirus RNA detected by RT-PCR methods. Four samples (2%) could not be assigned both G and P type specificity (non-typeable samples). G1P[8] represents the predominant genotype (32%) circulating in southern Benin, followed by the G2P[4] (26%), G3P[6] (16%) and G12P[8] (13%). Other uncommon genotypes in Benin such as G9P[8] (3%), G1P[6] (3%), G3P[8] (2%), G2P[8] (1%), G2P[6] (1%), G12P[6] (1%), G9P[4] (1%) and mixed genotypes (1%) were detected, albeit at low rate (Fig. 1a).

Distribution of genotypes by gender and age in Benin: There was greatest variability of genotypes in children aged from 0 to 18 months. This variability was less after 18 months. Uncommon genotypes were most observed in children under 24 months of age (Fig. 1b). However, there were no significant differences in genotype distribution when genders were compared (p > 0.05).

#### Distribution of genotypes in Cotonou

From the 330 samples collected, 147 (44.6%) contained detectable rotavirus RNA and 144 were genotyped for G and P. The common genotype combinations were G1P[8] (30%) followed by G2P[4] (23%), G3P[6] (16%), and G12P[8] (15%) (Fig. 1c). Three (2%) stool samples were non-typeable for G and P and only one (1%) sample showed mixed genotype. Less common genotypes such as G1P[6] (4%), G9P[8] (3%), G3P[8] (2%), G2P[8] (1%) G2P[6] (1%), G12P[6] (1%) and G9P[4] (1%) were detected.

There is great variability of genotypes in children aged from 0 to 18 months. This variability is less after 18 months (Additional file 1: Figure S1b).

#### Distribution of genotypes in Abomey-Calavi

In Abomey-Calavi, 39 (43.3%) stool samples were positive by RT-PCR. G1P[8] and G2P[4] genotype combinations were the most common (38% and 36% respectively), followed by G3P[6] (15%), G12P[8] (5%). No mixed genotypes were found and one sample was untypeable (Additional file 1: Figure S1c).

The greatest variability of genotypes was more observed in children less than 18 months in Abomey-Calavi. (Additional file 1: Figure S1d).

### Discussion

In the two localities that hosted the study, G1P[8] was the prevalent genotype, accounting for 38% in Abomey-Calavi and 30.0% in Cotonou or 32.0% across the two sites. According to a systematic review [17] describing the epidemiological situation in Africa, G1P[8] similarly was the predominant genotype with an overall prevalence of 22.64%. Taking into account the five regions of Africa (West, East, Central, North and South), the rate obtained varied from one region to another with a higher prevalence in North Africa (37.10%), while it was 14.35% in the West Africa [17].

At country level in West Africa, before vaccine introduction, Ghana reported G1P[8] (20%) as the predominant genotype [18], but others genotypes such as G6P[6] was found to be medically important in Burkina Faso [19], G12P[8] in Côte d’Ivoire [20] and G4P[8] and G12P[8] in Nigeria [21, 22]. During the post vaccine period, Ghana reported G1P[8] as the fourth common genotype detected with a rate of 8.0% [18]. Thus, there was a decrease in the prevalence rate and a replacement of strains after the introduction of the vaccine. Nearly similar observations are made in Zambia where G1P[8] initially reported as the predominant genotype (49%) in 2008 during the pre-vaccine period remained predominant the first year after vaccine introduction at 25% but changed in subsequent years [23]. On the other hand, according to the study of Seheri et al. [24], in Eastern and Southern African countries before and after vaccine introduction, data from six countries showed no difference in strains circulation during the pre- and post-vaccine introduction eras. So, it is not conclusive that after vaccine introduction there is a switch of strains due to vaccine pressure or this is just an annual fluctuation of strains.

In systematic review by Ouermi et al. [17] showed that G1P[8] was the most predominant genotype, followed by G2P[4], G9P[8] and G2P[6]. Similarly, in our study, G2P[4] proved to be the second-most prevalent genotype circulating in the south of the country where the study was conducted. However, in Niger and the Democratic Republic of Congo, G2P[4] was the predominant genotype before vaccine introduction [25, 26]. However, G2P[4] was found circulating in the Central Africa Republic at a rate of 13%, in Cameroon at 5.9%, Burkina Faso at < 1% and Gabon at < 2% [27–30].

G12P[8] is the second emerging genotype, detected for the first time in Philippines in 1987, in children under 2 years of age [31]. Mainly limited to Asian countries, G12P[8] has been reported worldwide over the last 20 years [32].

Commonly detected at low rates in Africa, over the past decade, G12P[8] has been isolated as a predominant genotype in some countries such as Côte d'Ivoire and Nigeria in the pre-vaccination period [20, 22], and Ghana in the post-vaccination period [18]. Also, according to some studies, G12P[8] may come from a reassortment between human and pig rotavirus strains [32]. The presence of this genotype in our study without any pre-existing vaccination context, strongly suggests that G12P[8] appearance in Benin, is due to natural rotavirus genotype occurrence, not vaccine pressure.

On the other hand, G9P[8] recognized as being the fifth-most prevailing genotype [10, 12] was found in our study at a rate of 3%. This genotype seems to be more present in East and North Africa [33, 34]. Even at this relatively low level, it deserves to be monitored in Benin since it was one of the emerging genotypes of the last 20 years [35].

During the study 2% (4/186) of the samples were untypeable for G or P, which is less than the rate of untypeable samples previously reported from Sub-Saharan countries (8.6–14.6%) [36]. This low level of untypeable G/P genotypes could be explained by the technique used which incorporates more recently designed primers.

Unlike reported by the African Rotavirus Surveillance Network where mixed genotypes accounted for 12–14% [36], in our study, a single sample (1%) showed a mixed genotype. Indeed, close association of humans with domesticated animals in most countries of the developing region lead to gene reassortment events within commonly circulating animal rotavirus strains and thus give rise to a large genomic diversity and frequent occurrence of mixed infections [20]. However, studies from some Africans settings have shown no mixed genotypes [37] or a very low rate (1%) of mixed infections [20] similar to our findings. Also, as suggested by comments from the works of Boula and colleagues, the incorporation and use of new and updated one step multiplex genotyping assays in this study could strongly explain the low rate of mixed genotypes.

Atypical rotavirus G and P combinations were detected at low frequency (2%) similar to the rate described previously by Seheri et al. [24]. No correlation between age group and genotype was observed in this study, although it has been noted a higher variability of genotypes in children less than 18 months. This observation could probably due to the fact that more samples came from the youngest age groups.

It is important that further studies be conducted to determine the true intrinsic determinants of occurrence of rotavirus gastroenteritis in children. Studies on the genetic differences in histo-blood group antigens in our populations (secretor, non-secretor, Lewis antigens) could help to learn more about rotavirus epidemiology.

## Limitations

The limitations of this study are due to the fact that samples came from the southern part of Benin where the distribution of genotypes could differ from that of the northern region due to the difference in seasons and the living behavior of the populations between the two regions.

## Supplementary information

**Additional file 1: Table S1.** Distribution of the study population by age and gender. **Figure S1a.** Sample collection sites. (1) Abomey Calavi in the Atlantic Region (a = Central Clinic of Abomey Calavi) and (2) Cotonou in the Central Region (b = Anastasis Hospital, c = Polyclinic St Vincent de Paul and d = Mênontin Hospital). **Figure S1b.** Distribution of genotypes by age in Cotonou. **Figure S1c.** Distribution of genotypes from 90 stool samples in Abomey-Calavi. **Figure S1d.** Distribution of genotypes according to age in Abomey-Calavi.

## Data Availability

The raw genotyping data including those in the supplementary table and figures are kept by the Centers for Disease Control and Prevention (CDC) and can be requested from Dr. Mathew Esona using email address: mdi4@cdc.gov.

## Abbreviations

EIA: Enzyme linked immunosorbent assay
NIP: National Immunization Programs
qRT-PCR: Quantitative reverse transcription-polymerase chain reaction
RT-PCR: Reverse transcription-polymerase chain reaction
WHO: World Health Organization
