# Supplementary material for: Molecular characteristics of rotavirus genotypes circulating in the south of Benin, 2016–2018
Source: BMC Res Notes. 2020 Oct 19;13:485. doi: 10.1186/s13104-020-05332-7 (PMC7574571; doi:10.1186/s13104-020-05332-7)

## Slide 1
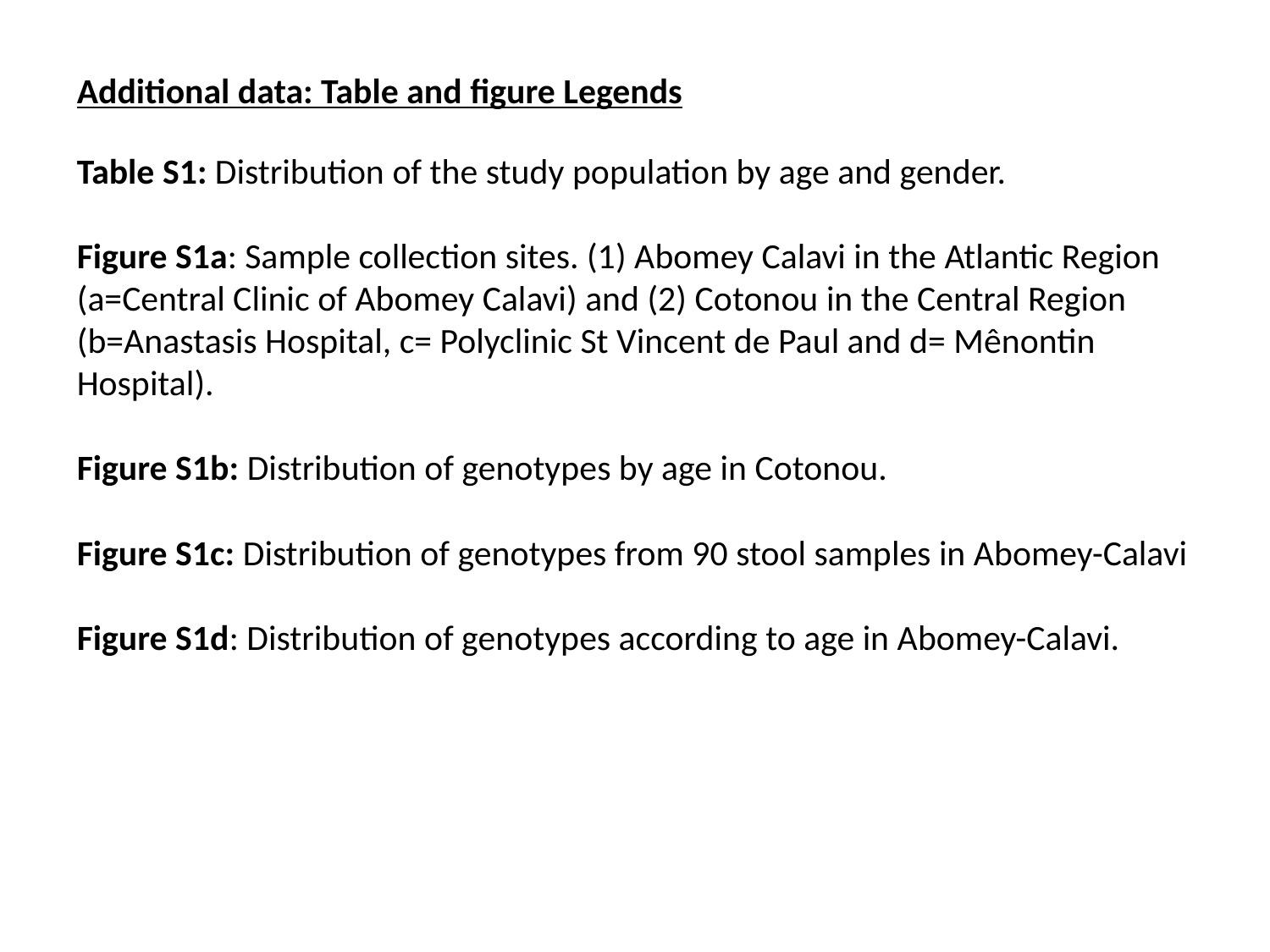

Additional data: Table and figure Legends
Table S1: Distribution of the study population by age and gender.
Figure S1a: Sample collection sites. (1) Abomey Calavi in the Atlantic Region (a=Central Clinic of Abomey Calavi) and (2) Cotonou in the Central Region (b=Anastasis Hospital, c= Polyclinic St Vincent de Paul and d= Mênontin Hospital).
Figure S1b: Distribution of genotypes by age in Cotonou.
Figure S1c: Distribution of genotypes from 90 stool samples in Abomey-Calavi
Figure S1d: Distribution of genotypes according to age in Abomey-Calavi.

## Slide 2
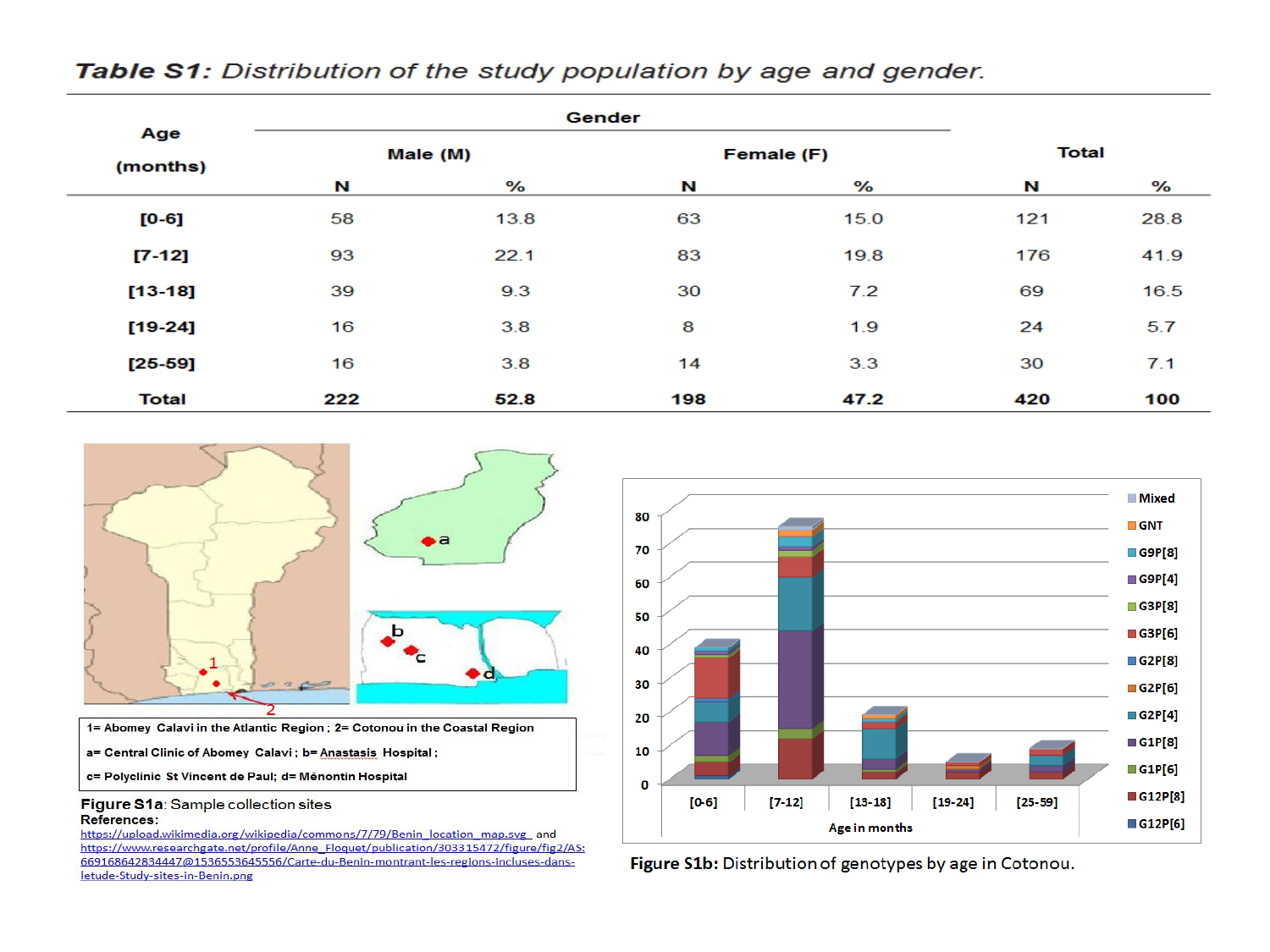

## Slide 3
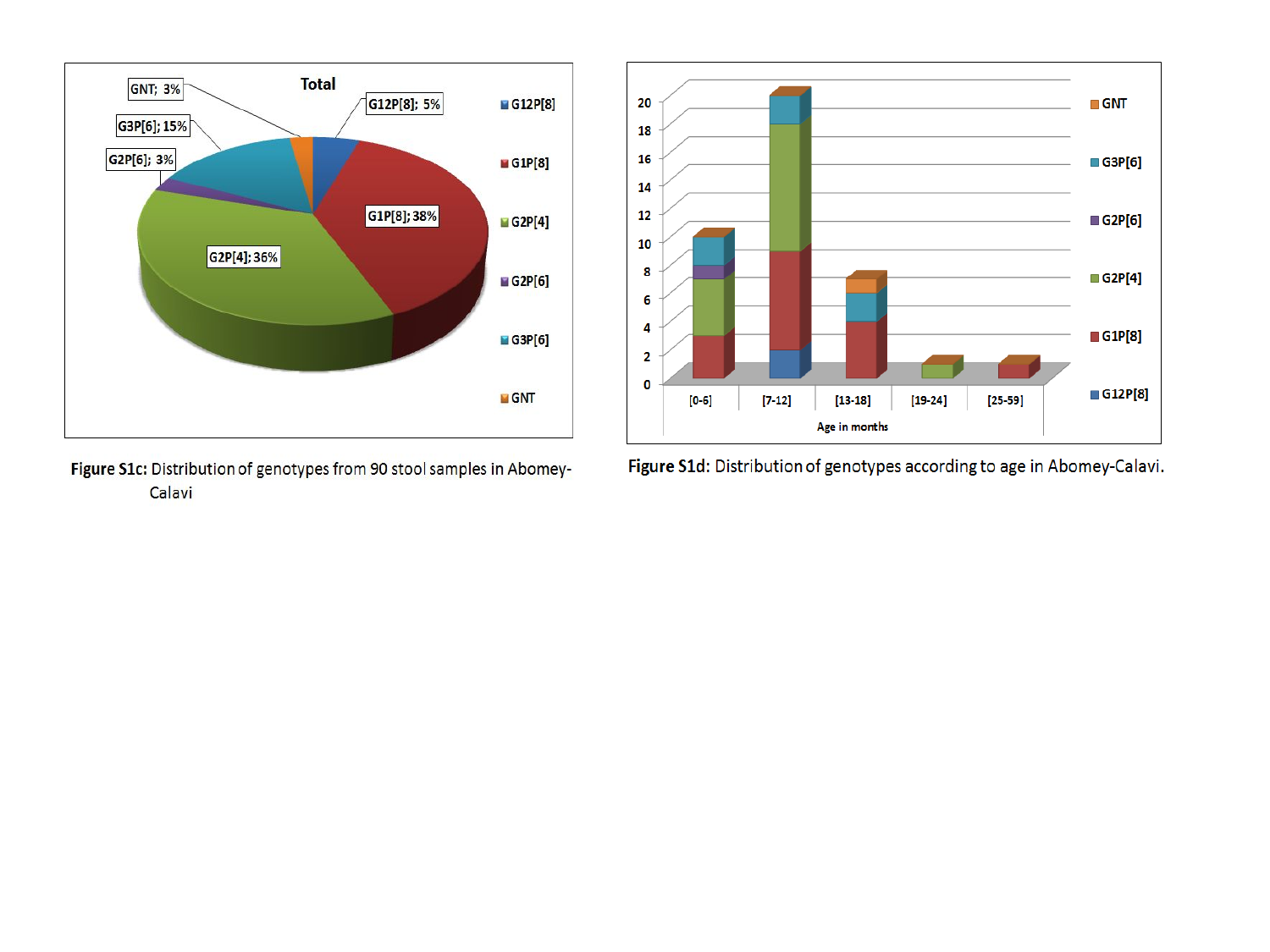

Supplement: Supplementary file 1 — Additional file 1: Table S1. Distribution of the study population by age and gender. Figure S1a. Sample collection sites. (1) Abomey Calavi in the Atlantic Region (a = Central Clinic of Abomey Calavi) and (2) Cotonou in the Central Region (b = Anastasis Hospital, c = Polyclinic St Vincent de Paul and d = Mênontin Hospital). Figure S1b. Distribution of genotypes by age in Cotonou. Figure S1c. Distribution of genotypes from 90 stool samples in Abomey-Calavi. Figure S1d. Distribution of genotypes according to age in Abomey-Calavi. [file 13104_2020_5332_MOESM1_ESM.pptx]
